# Research on Cryogenic Technology for Phased Array Feed Receivers with Multiple Refrigerators

**DOI:** 10.3390/s26196303

**Published:** 2026-10-06

**Authors:** Kai Wang, Liang Cao, Jun Ma, Hao Yan, Xiao-Fei Li, Jia-Hui Li, Mao-Zheng Chen

**Affiliations:** 1 Xinjiang Astronomical Observatory, Chinese Academy of Sciences, Urumqi 830011, China; wangkai@xao.ac.cn (K.W.); caoliang@xao.ac.cn (L.C.); majun@xao.ac.cn (J.M.); lixf@xao.ac.cn (X.-F.L.); lijiahui@xao.ac.cn (J.-H.L.); chen@xao.ac.cn (M.-Z.C.); 2 Xinjiang Key Laboratory of Microwave Technology, Urumqi 830011, China; 3 University of Chinese Academy of Sciences, Beijing 100049, China

**Keywords:** radio telescope, receiver, phased array feed (PAF), refrigeration, structural design

## Abstract

Similar to cryogenic receivers equipped in radio telescopes, Phased Array Feed (PAF) receivers can also achieve noise temperature improvements by refrigerating key components. This paper focuses on a cryogenic PAF receiver with a larger refrigeration capacity requirement, adopting a technical approach of full-array refrigeration and employing a cryogenic system consisting of multiple refrigerators for experimental investigation. The initial design adopted a structure where two M350 refrigerators were jointly used for refrigeration, and the measured temperature at the Low Noise Amplifier (LNA) mounting structure was only 22.3 K. Subsequently, the individual refrigeration performance of each refrigerator and the actual refrigeration power of each cold stage were tested and analyzed, and an improved partition design was proposed. In this design, the original LNAs and their mounting structure were divided into two equal parts, each independently refrigerated by one refrigerator. The improved partition design eliminated the thermal coupling and thermal back-flow in the common structure of the original joint refrigeration scheme, while halving the original thermal load and distributing it independently to each refrigerator, thereby enhancing the refrigeration efficiency of the entire cryogenic system. With this improved design, the measured temperatures of the two separated LNA mounting structures reached 15.2 K and 18.2 K, respectively, both achieving the target below 20 K. The above research systematically verifies the impact of structural differences on refrigeration performance in multi-refrigerator PAF cryogenic designs, providing a feasible approach for improving receiver noise performance, while also offering valuable guidance for the design of cryogenic systems composed of multiple refrigerators under large refrigeration capacity requirements.

## 1. Introduction

Radio telescopes are observational instruments in the field of radio astronomy [1]. Currently, several Chinese astronomical observatories are constructing large-aperture radio telescopes to enhance observational sensitivity, such as the QiTai 110 m radio telescope (Qi Tai Telescope, QTT) [2]. The receiver system is the signal reception device installed at the focal position of a radio telescope [3,4]. Compared with traditional multibeam receivers [5], the Phased Array Feed (PAF) receiver with leveraging beamforming algorithms and element multiplexing can generate multiple overlapping beams in the far field, thereby broadening the field of view of the radio telescope and further improving observational efficiency [6,7,8,9,10,11,12]. For instance, the prototype of the 19-element PAF receiver was developed by the 54th Research Institute of China Electronics Technology Corporation (CETC) for FAST (Five-hundred-meter Aperture Spherical radio Telescope), as described in Refs. [13,14].

One of the most critical performance metrics of a radio telescope system is sensitivity, and a larger receiving area from a bigger reflector, wider receiver operating bandwidth, and lower noise temperature all significantly enhance observation sensitivity [15]. As the core signal reception device in a radio telescope system, the receiver’s noise temperature is directly linked to sensitivity [16]. The lower the receiver noise temperature is, the higher the observation sensitivity is, meaning the entire observing system can detect fainter radio signals. Generally, the thermal noise generated by the receiver is referred to as its noise temperature, which is equivalent to the calibrated value of the receiver’s intrinsic intensity response. Similar to high performance cryogenic receivers, PAF receivers can also achieve lower noise temperature by cooling key components such as Low Noise Amplifiers (LNAs) and antenna array [17,18,19].

The article is organized as follows: Section 1 provides an introduction to the relevant technical background. Section 2 focuses on the analysis and evaluation of cryogenic schemes for PAF receivers, including the analysis of two representative cases and the evaluation of PAF receiver noise temperature under different cryogenic schemes. Section 3 presents the specific cryogenic PAF receiver structural design and refrigeration experiments carried out in this paper. Section 4 discusses the relevant physical mechanisms, the relationship between thermal analysis and refrigeration capacity, as well as the impact of LNA power consumption on refrigeration efficiency. Section 5 summarizes the research work of this paper and provides an outlook for future work.

## 2. Cryogenic Schemes for PAF Receivers

### 2.1. Relevant Cryogenic Design Cases

#### 2.1.1. Partial Refrigeration Scheme

The National Radio Astronomy Observatory (NRAO) initiated PAF development efforts for the large-aperture Green Bank Telescope (GBT). Subsequently, Brigham Young University joined the research team, and they worked together on developing an L-band focal plane array capable of cooling LNAs, known as the Focal L-band Array for GBT (FLAG) [20,21,22]. Compared with earlier ambient temperature PAF designs, FLAG represents a milestone in development as the first cryogenic PAF receiver. As shown in Ref. [23], FLAG employs independent dual-polarized dipole elements, with the LNAs connected behind the elements designed to operate within a cryogenic Dewar to achieve lower noise temperatures.

Compared to ambient temperature PAF receivers, FLAG adopted the refrigeration scheme that only cooled the LNAs. This scheme achieved significant improvements in noise temperature, laying the groundwork for later to refrigerate the entire array.

#### 2.1.2. Full-Array Refrigeration Scheme

Based on the development experience with FLAG, Brigham Young University in collaboration with Cornell University continued to develop a 19-element fully refrigerated PAF prototype for the Arecibo radio telescope, named the AO19 Cryo-PAF [24,25,26]. As shown in Ref. [27], the prototype adopts dual-polarized dipole elements arranged in a hexagonal layout. To achieve full-array refrigeration, the AO19 design embeds the array within a Dewar, with a layer of ROHACELL foam surrounding the array to provide thermal insulation and structural support.

Compared to the refrigeration scheme of FLAG, A019 adopted the refrigeration scheme that cooled the LNAs and array. This scheme achieved further improvements in noise temperature and has since become the commonly adopted cryogenic solution for pursuing high noise performance in PAF receivers.

### 2.2. Noise Temperature Estimation for Cryogenic PAF Receivers

#### 2.2.1. 20 cm Band PAF Receiver

**Figure 1:**
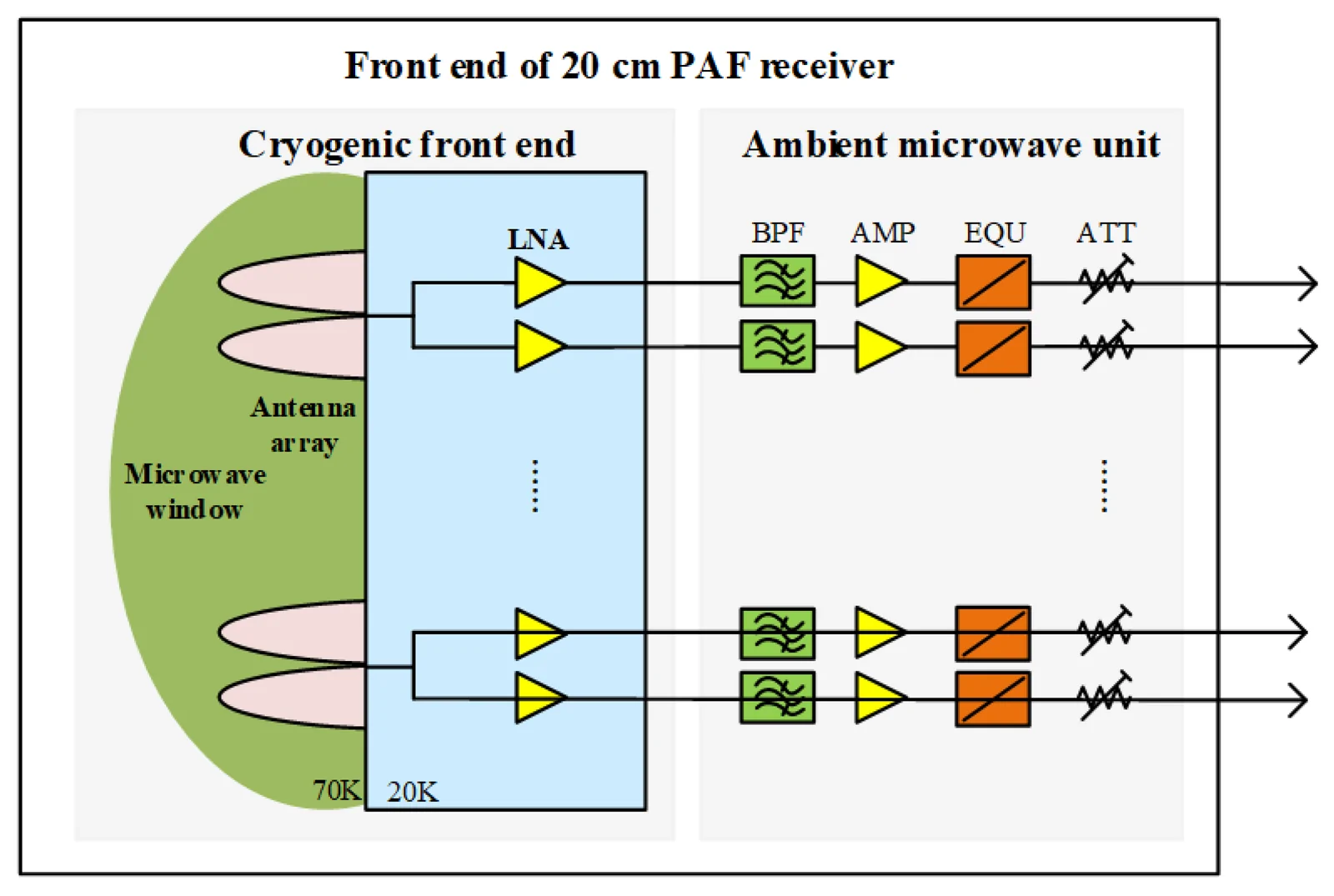
Front-end configuration of the 20 cm band PAF receiver. The cryogenic Dewar is equipped with a microwave window to enclose the antenna array and the LNAs, forming the cryogenic front end of the PAF receiver, with the aim of achieving a lower noise temperature. After being output from each LNA, the signals pass through the ambient temperature microwave unit of the PAF receiver, which consists of components such as band-pass filters (BPFs), amplifiers (AMPs), equalizers (EQUs), and programmable attenuators (ATTs), to process each path of signal and deliver them to the downstream digital backend.

The 20 cm band PAF receiver planned for the QTT will operate over a frequency range of 0.7–1.8 GHz, features dual-linear polarization with no fewer than 96 elements per polarization, and a target noise temperature for the receiver is ≤25 K. To achieve such stringent noise temperature performance while meeting the above bandwidth and other specifications, it is necessary not only to cool the LNAs but also to adopt a refrigeration scheme for the full array. The front-end configuration of the 20 cm band PAF receiver is shown in Figure 1.

#### 2.2.2. Noise Temperature Estimation

As shown in Figure 1, the primary contributors to the noise temperature of the PAF receiver are the microwave window (radome) loss, the antenna array loss, the coupling noise of the antenna array, and the noise contribution of the LNAs.

Referring to the partial and full-array refrigeration designs described in Section 2.1, we evaluated the noise temperature of the PAF receiver under both schemes. The insertion losses of the microwave window (radome) and the array used in the evaluation were obtained from simulations, the array coupling coefficients were estimated based on engineering measurements, and the noise temperature (noise figure, 0.7 GHz) of the LNAs was derived from the manufacturer’s specifications at ambient temperature (300 K) and cryogenic temperature (15 K). Table 1 and Table 2 present the estimated noise temperatures of the PAF receiver when the antenna array is at ambient temperature (295 K) and cryogenic temperature (77 K), with the LNAs at cryogenic temperature (20 K).

From the above calculations, it can be seen that when the LNAs are refrigerated to a physical temperature of 20 K, the corresponding noise temperatures of the PAF receiver are 51.6 K and 22 K for the array at ambient and cryogenic, respectively. Therefore, for the development of PAF receivers with stringent noise requirements, in addition to refrigeration of the LNAs, full-array refrigeration is also a necessary scheme.

## 3. Cryogenic PAF Receiver Structural Design and Refrigeration Experiments

### 3.1. Structural Design for Full-Array Refrigeration

#### 3.1.1. Vivaldi Antenna Array Prototype

**Figure 2:**
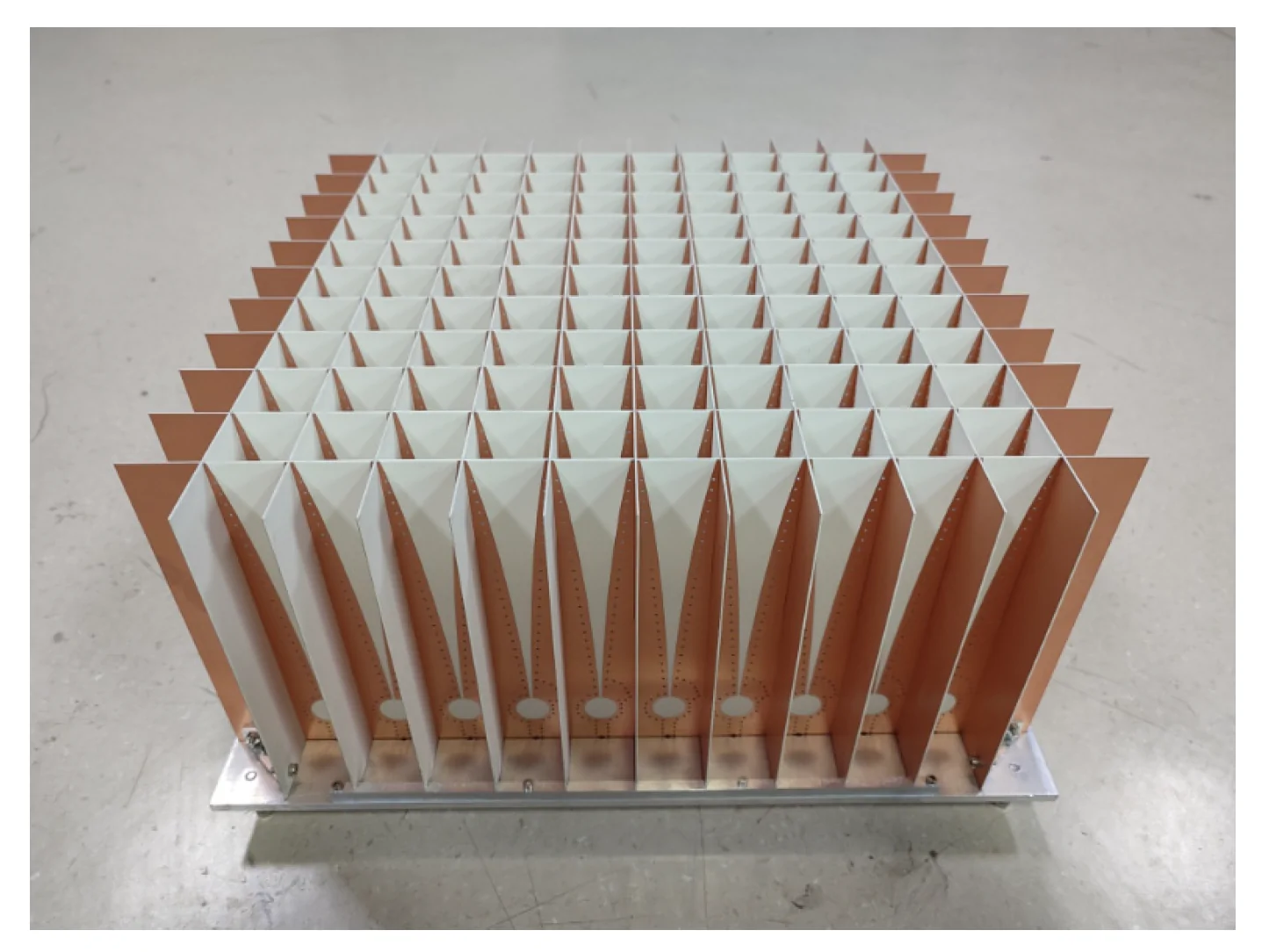
Vivaldi antenna array prototype. This array is designed to operate over a frequency range of 0.7–1.8 GHz, adopting a PCB-based dual-linear polarization design. The elements are arranged in a rectangular 10 × 11 layout with the element spacing of 30 mm.

The Microwave Technology Laboratory of Xinjiang Astronomical Observatory (XAO) has successfully developed a Vivaldi antenna array prototype, as shown in Figure 2. After the preliminary microwave performance testing and verification, this array can be used for the cryogenic design and validation of a PAF receiver.

#### 3.1.2. Initial Cryogenic Structure Validation

**Figure 3:**
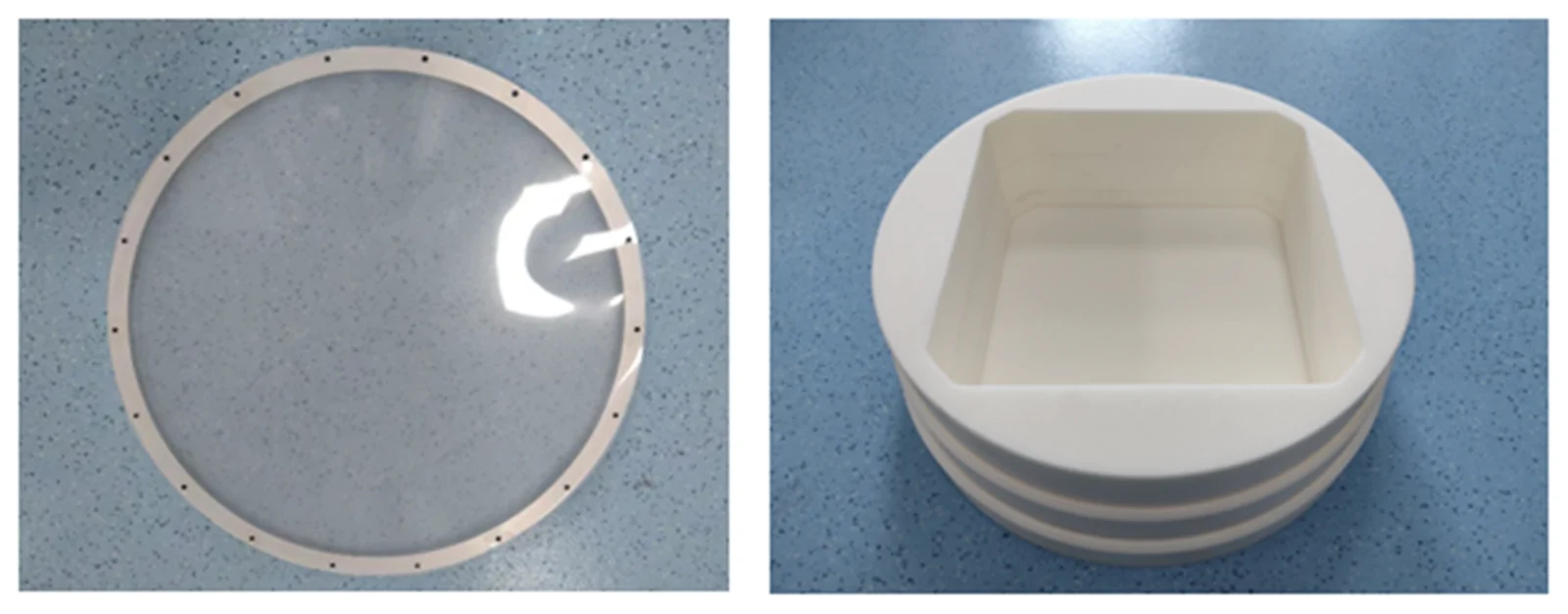
Composition of the microwave window. The outermost layer of this window is made of polyester film (Mylar), which is secured to the top of the Dewar by a ring-shaped metal clamp for sealing and wave-transmitting functions. Beneath the polyester film, a rigid foam made of polymethacrylimide (PMI) is employed, consisting of three bonded layers with the central space hollowed out to accommodate the Vivaldi antenna array.

We adopted the same scheme of full-array refrigeration design as the AO19. Figure 3 shows the large-aperture microwave vacuum window required for full-array refrigeration. The window is composed of a sealing film and a supporting and wave-transmitting foam.

**Figure 4:**
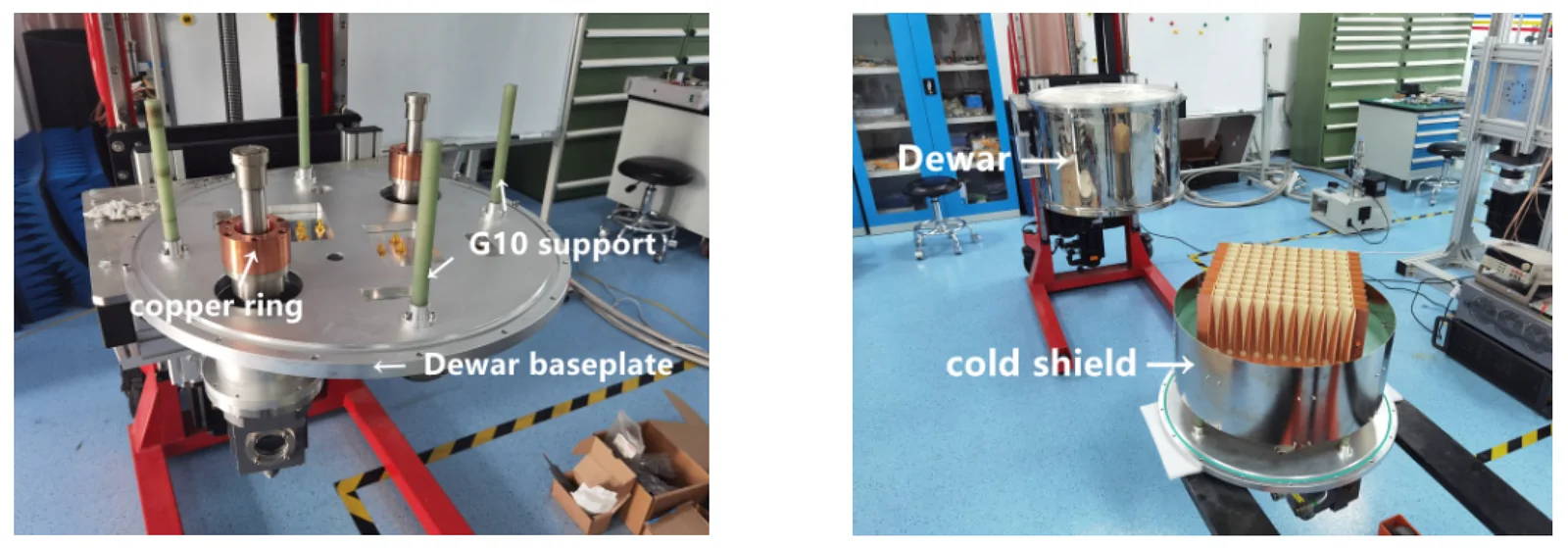
Dewar and its associated structures. The 77 K cold stages of two M350 refrigerators are each fitted with a copper ring. The upper side of each copper ring is connected to the cold shield baseplate for heat transfer. Four G10 support legs pass through the cold shield baseplate to support the array and provide thermal insulation. After the cold shield and the antenna array are assembled, they are integrated with the microwave window and then the entire assembly is fitted inside the Dewar.

In addition to the large-aperture microwave vacuum window, the initial cryogenic structure also included a cylindrical Dewar, an inner cold shield, and a Dewar baseplate with the refrigerators and support structures mounted on it, as shown in Figure 4. These components enclosed the antenna array to be cooled within the Dewar cavity, fulfilling the functions of thermal insulation, sealing, structural support, and heat transfer.

**Figure 5:**
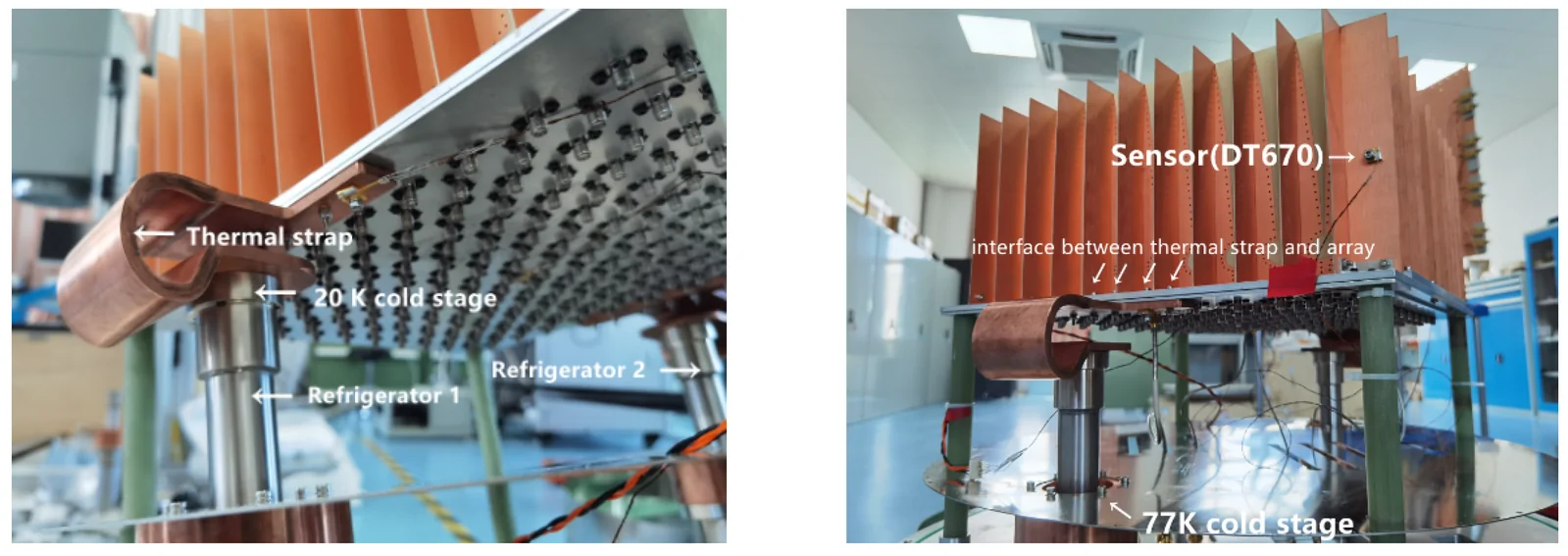
Initial cryogenic structural design. The experiment selected a Lake Shore DT-670 silicon diode cryogenic temperature sensor, configured with a BO package and four-wire measurement. The measured temperature at the array antenna was 58 K.

At the time of the initial PAF cryogenic experiment, the post-array LNAs were not yet available, so the primary goal of the first design was to refrigerate the antenna array to below 20 K. Therefore, in addition to the external Dewar and cold shield design, the core of the initial design was to jointly connect the 20 K cold stages of two refrigerators to the array mounting backplane via their respective thermal straps, as shown in Figure 5.

Since the initial cryogenic structure design failed to refrigerate the array to below 20 K, we performed a thermal analysis of this structure in Ref. [28]. Based on the capacities of the two cold stages of the M350 refrigerator (20 W @ 77 K, 4 W @ 20 K), the analysis indicated that the large-aperture microwave window, which had partial contact with the top of the array, contributed significant conductive heat leakage, resulting in a total refrigeration demand far exceeding the power of the 20 K cold stage. Given the limited refrigeration capacity of the 20 K cold stage, the core priority of the cryogenic PAF design should be to ensure that multiple LNAs can be cooled to 20 K, rather than mandating that the antenna array also reach this temperature. Therefore, we adjusted the cryogenic structure based on the subsequently procured LNAs.

#### 3.1.3. Updated Cryogenic Structural Validation

**Figure 6:**
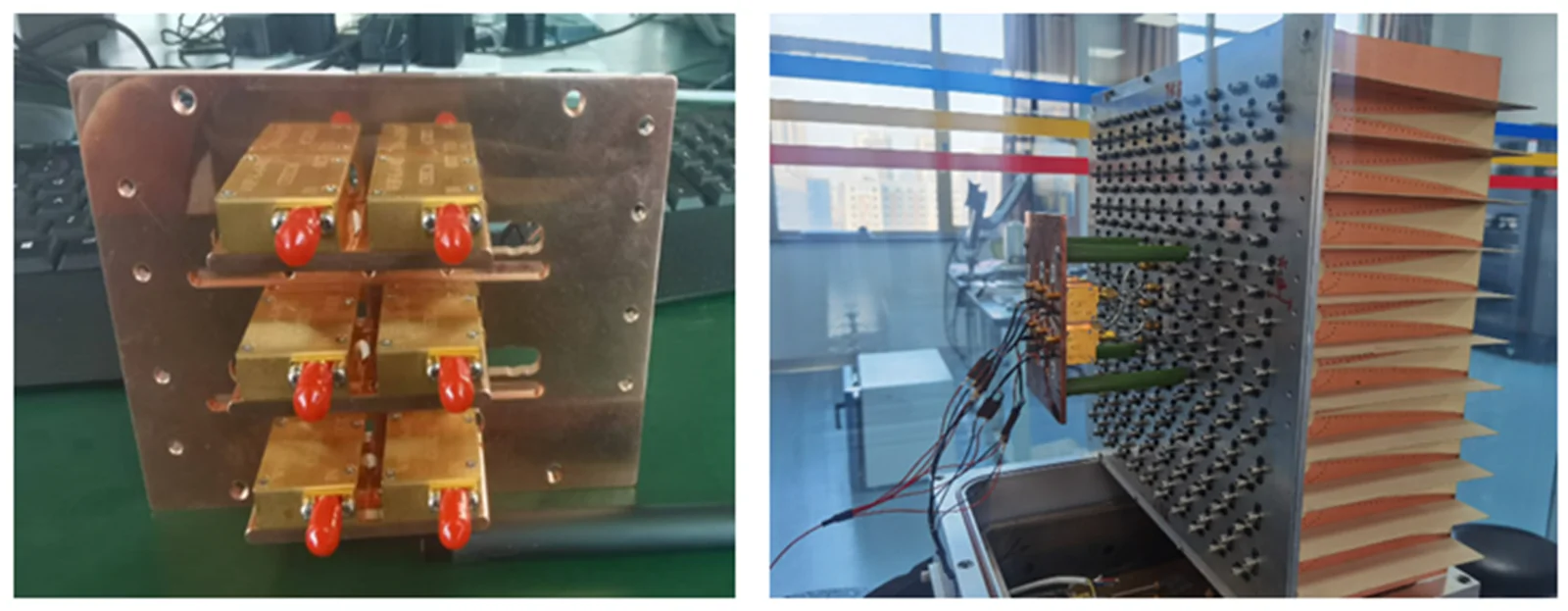
Assembly of LNAs. The cryogenic LNAs assembled behind the array antenna, mounted onto a copper fixing and thermal conduction backplane, with the entire assembly secured to the rear of the array by four epoxy-fiber glass rods for convenient cable connection.

After the initial cryogenic structure validation, we procured six cryogenic LNAs. Considering that the LNAs will also generate heat and consume power after being turned on, our design involved installing them on a copper structure with good thermal conductivity and fixing them on the back side of the central array, facilitating connection to the central array elements, as shown in Figure 6.

**Figure 7:**
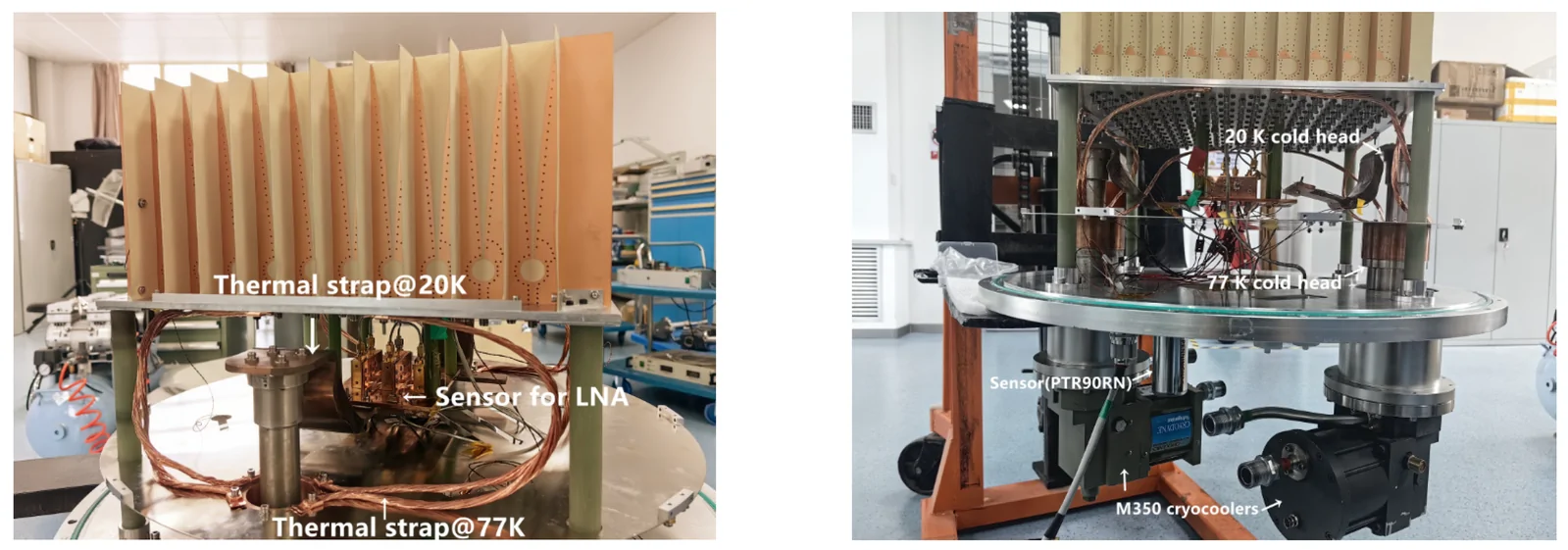
Updated cryogenic structural design. The 77 K cold head is connected to the array backplate via multiple thermal straps, and is also directly connected to the cold shield through a copper ring, ensuring that the mechanical structures requiring large refrigeration capacity are all maintained at the 77 K cold stage. Meanwhile, the 20 K cold head is connected to the LNA mounting structure via a thermal strap, ensuring that the LNAs can be cooled to 20 K.

Based on the refrigeration capacities of the M350 for two cold stages and the validation analysis of the initial cryogenic structure, considering that the 20 K cold stage has only limited refrigeration capacity, it is used solely to cool the LNAs of the PAF receiver in the new cryogenic structure, while the 77 K cold stage with greater cooling capacity is used to cool the antenna array and the cold shield, as shown in Figure 7.

**Figure 8:**
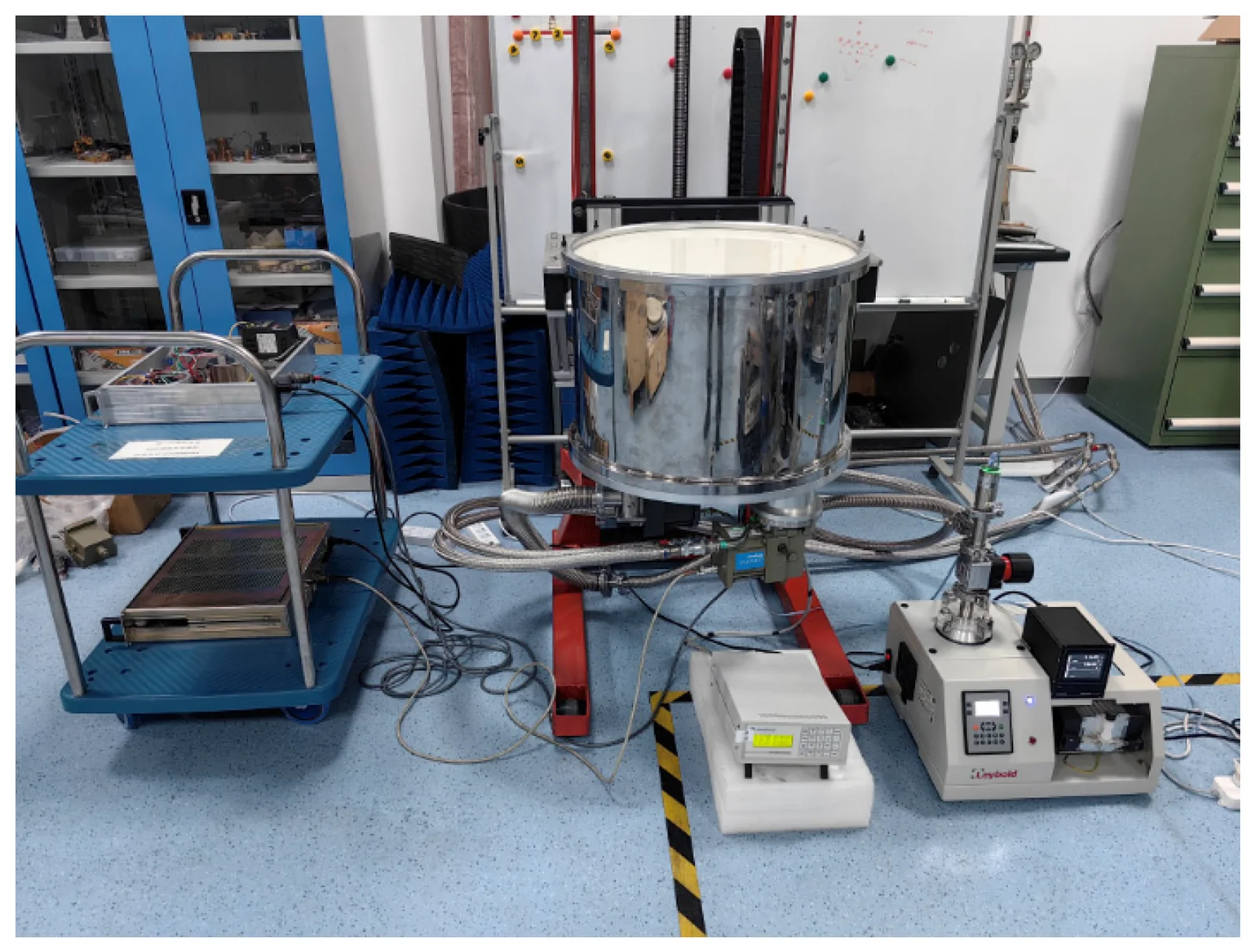
Refrigeration experiment.

Based on the current cryogenic design employing two M350 refrigerators operating jointly, we conducted a refrigeration experiment on the assembled cryogenic prototype, as shown in Figure 8. The measurement results showed that the vacuum for the cryogenic Dewar of the prototype was 1.8 × 10^−5^ Pa, and the refrigeration temperature of the LNA mounting structure was 22.3 K.

Based on the thermal analysis of the initial cryogenic structure, the refrigeration capacity requirement at the 77 K cold stage for the current updated cryogenic structural design is approximately 32.6 W, and the 20 K cold stage is 3.9 W (including the power dissipation of the six installed LNAs). Therefore, the currently selected two M350 refrigerators (20 W @ 77 K × 2, 4 W @ 20 K × 2) can theoretically meet the cooling requirements of both cold stages, particularly ensuring that the temperature at the LNA mounting structure remains below 20 K, rather than only achieving 22.3 K.

### 3.2. Structural Improvement for Full-Array Refrigeration

#### 3.2.1. Cryogenic Test with a Single Refrigerator

**Figure 9:**
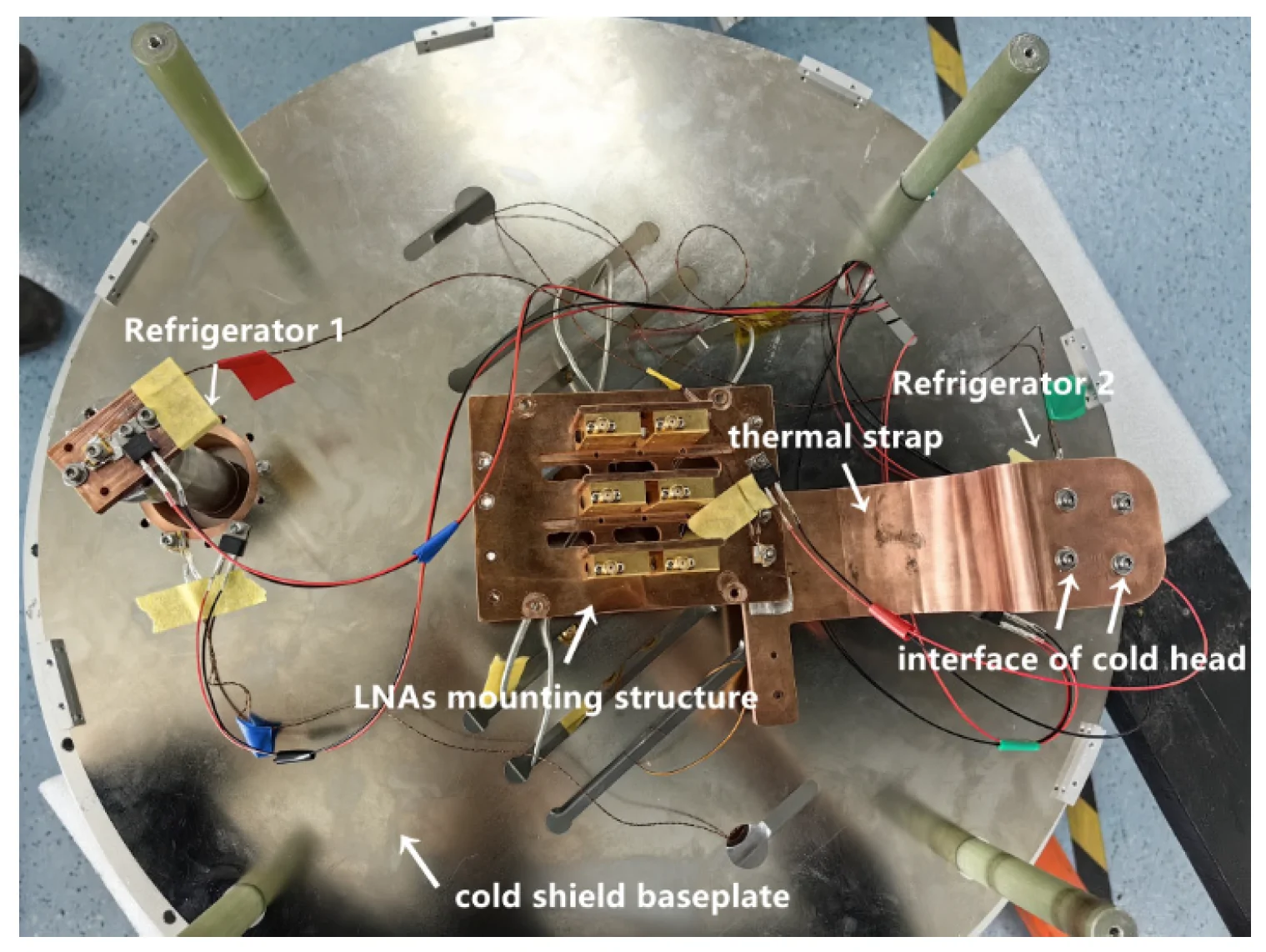
Refrigeration of the LNA mounting structure using refrigerator 2. The measured results showed that the refrigeration temperature at the LNA assembly was 22.1 K with refrigerator 1 and 22.7 K with refrigerator 2. Compared with the 22.3 K achieved when both refrigerators were operated together, these results show minimal variation, indicating that there is no significant difference between joint refrigeration with two refrigerators and individual using a single one.

First, based on the current cryogenic design employing two M350 refrigerators for joint refrigeration, we performed an experiment where each refrigerator was used independently. Specifically, refrigerator 1 (left) and refrigerator 2 (right) were used separately to refrigerate the LNA mounting structure, and Figure 9 shows the configuration where only the 20 K cold head of refrigerator 2 (right) is connected to the LNA assembly via a thermal strap.

#### 3.2.2. Refrigeration Power Measurement of the Refrigerator

**Figure 10:**
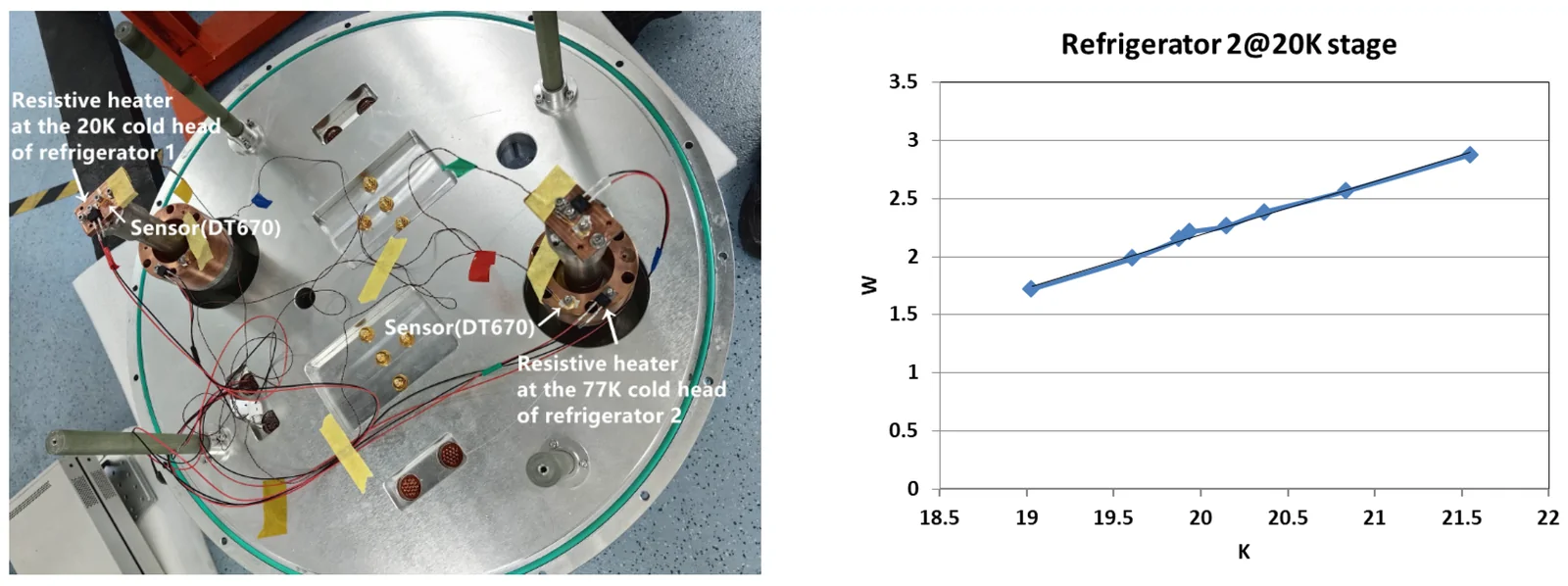
Measurement of actual refrigeration power of two refrigerators. The measurement results showed that the actual refrigeration capacity of refrigerator 1 (**left**) was 3.02 W @ 20 K and 18.4 W @ 77 K, while that of refrigerator 2 (**right**) was 2.21 W @ 20 K and 14.4 W @ 77 K. The key finding is that the 20 K cold stages of both refrigerators failed to achieve the nominal power of 4 W.

Considering that the two M350 refrigerators used in the cryogenic prototype were not brand new and may have suffered some degradation in refrigeration capacity (less than 4 W @ 20 K and 20 W @ 77 K), we measured the actual refrigeration power of both cold stages for each refrigerator. Specifically, a resistive heater was installed at each cold stage, as shown in Figure 10. After the refrigerator reached a steady state, we gradually increased the supply voltage to the resistive heater to raise the measured temperature of the cold head. The heating power at the moment when the cold head temperature rose to the rated temperature (20 K or 77 K) was recorded, and this value was taken as the actual refrigeration power of that cold stage. Meanwhile, we selected several measured temperature points around the nominal temperatures and recorded the corresponding heating power of the resistive heaters. By fitting these data, we obtained the relationship between temperature and refrigeration power in Figure 10. Based on this relationship and the independently measured temperature of Refrigerator 2 (22.7 K) shown in Figure 9, the refrigeration power required for the LNAs and their mounting structure was determined to be approximately 3.43 W.

**Figure 11:**
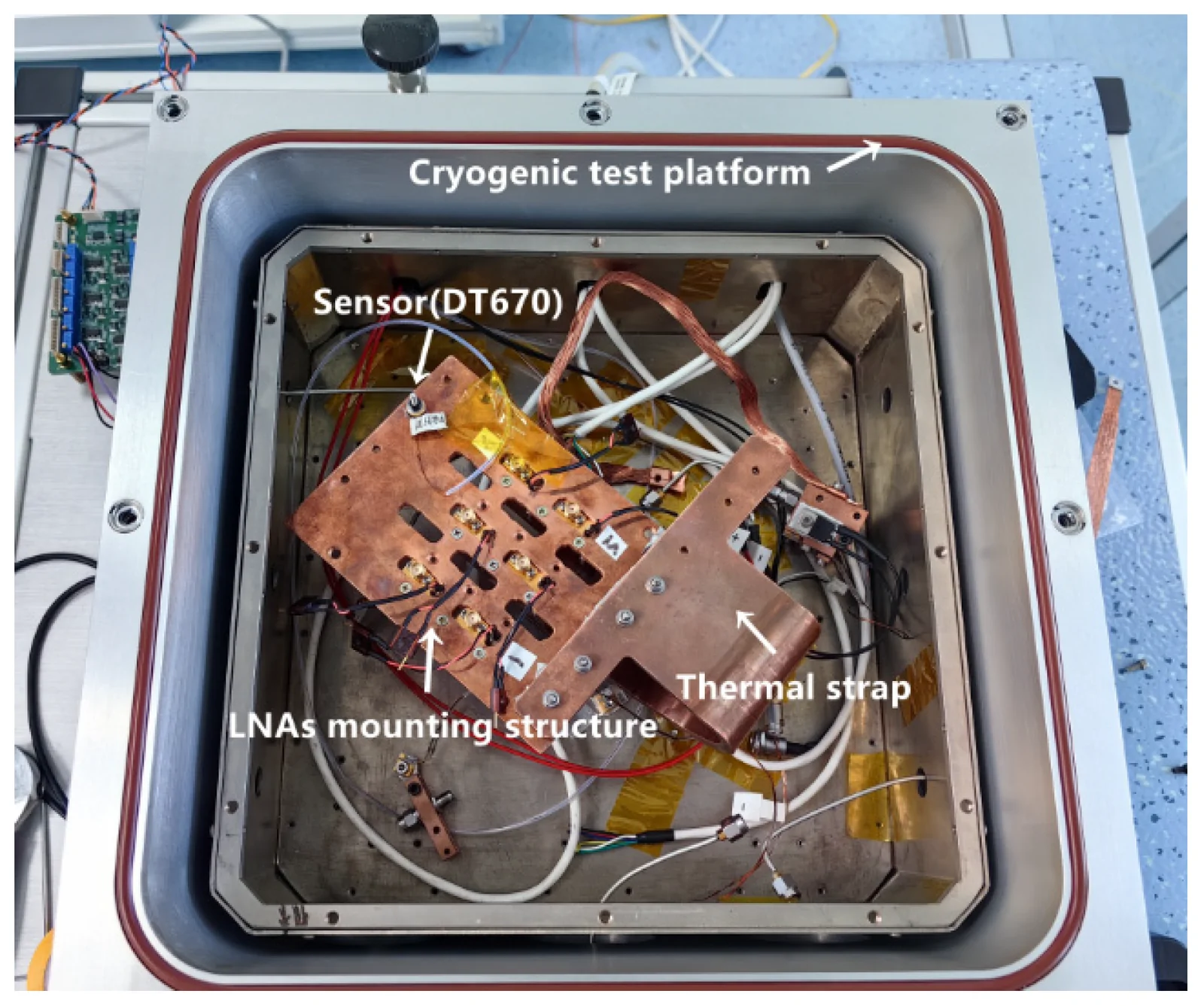
Refrigeration experiment of the LNA mounting structure under the cryogenic test platform. The measured temperature was below 20 K, demonstrating that the actual power demand of the LNA mounting structure is less than 4 W, which is consistent with the previously calculated theoretical cooling power requirement of 3.43 W.

We also conducted a refrigeration experiment on the LNA mounting structure using the cryogenic test platform (equipped with a single M350 refrigerator) at the Microwave Technology Laboratory of XAO, as shown in Figure 11. Prior to the test, we first verified using the resistive heater method that the 20 K cold stage of the M350 refrigerator used in this platform could achieve a power of 4 W. Under this cryogenic test platform, the LNA mounting structure was measured to be refrigerated to 14.5 K.

#### 3.2.3. Cryogenic Structural Improvement with Partition Design

**Figure 12:**
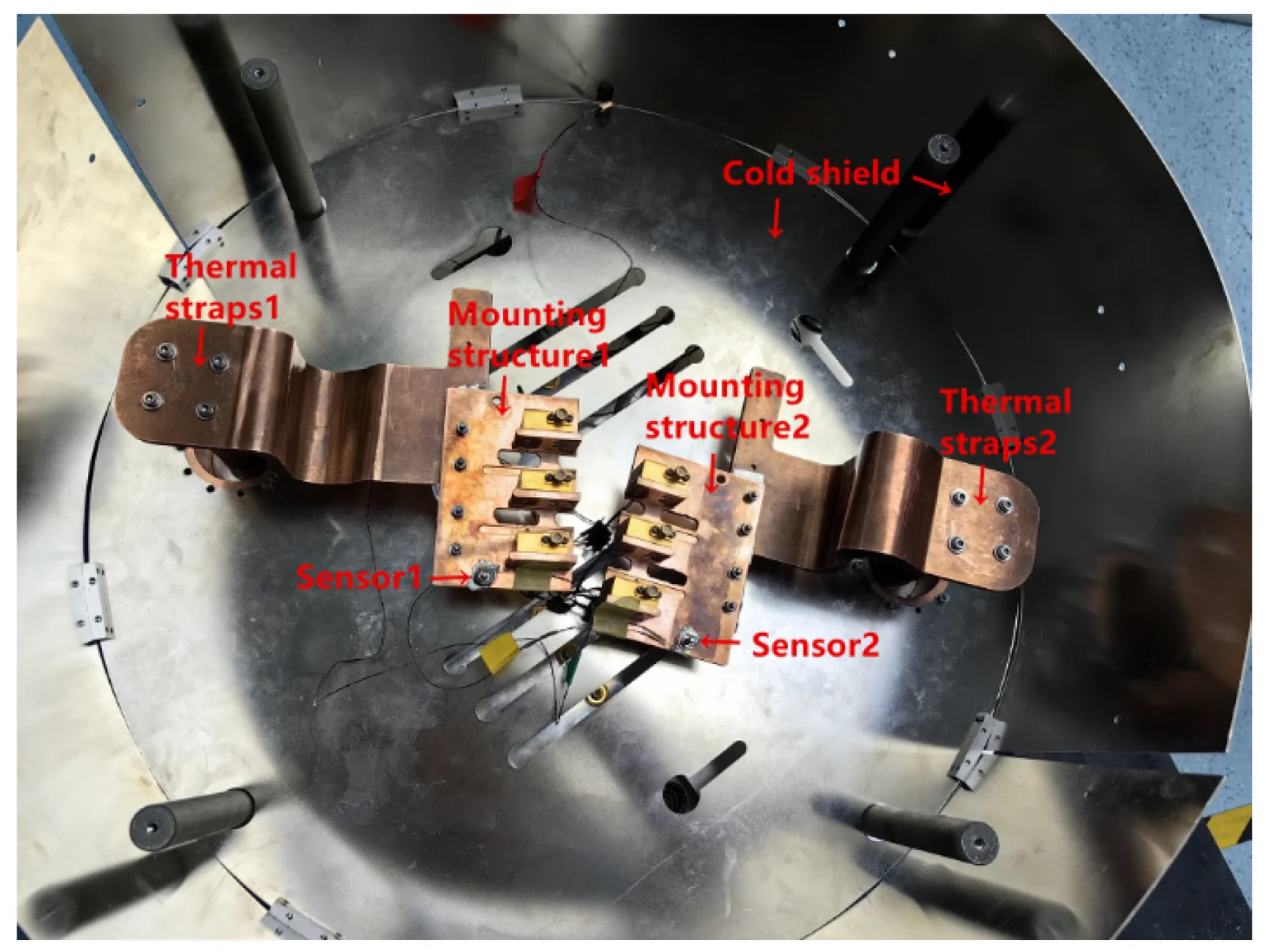
The 20 K cold stage cryogenic structure after the partition design. In addition to the original LNA mounting structure being divided into two equal parts, the number of LNAs, support structure and thermal strap corresponding to each refrigerator’s 20 K cold stage are identical. The measured temperature of the LNA mounting structure connected to refrigerator 1 was 15.2 K, and that connected to refrigerator 2 was 18.2 K, both achieving the target below 20 K.

Based on the measured refrigeration power of the 20 K cold stages of the two refrigerators in the cryogenic prototype (3.02 W and 2.21 W) and the calculated power demand of the LNA mounting structure (3.43 W), we proposed a partition design to improve the cryogenic structure. Specifically, the partition design involves dividing the original LNA mounting structure into two equal parts, with each refrigerator cooling one part. This effectively halves the refrigeration power demand on each refrigerator’s 20 K cold stage compared to the original configuration, thereby matching the measured power of each refrigerator’s 20 K cold stage. The improved LNA mounting structure and its assembly inside the Dewar are shown in Figure 12.

Finally, based on the actual measured temperature of the antenna array after the partition design (165 K) and taking the average temperature of the two measured LNA mounting structures (16.5 K), we estimated the equivalent noise temperature of the current cryogenic PAF. The estimation result shows that the overall noise temperature of the cryogenic PAF receiver is 33.7 K, as shown in Table 3.

## 4. Discussion

This paper addresses the cryogenic structure of a PAF receiver composed of multiple refrigerators, achieving improved refrigeration efficiency through a partition design. The core physical mechanism lies in cutting off the thermal coupling and thermal back-flow present in the original joint refrigeration structure. First, in the original joint structure, due to the inherent performance difference between the two refrigerators, refrigerator 1 exhibited stronger refrigeration capability with a lower standalone refrigeration temperature (22.1 K), while refrigerator 2 had slightly weaker capability with a higher temperature (22.7 K). When applied to the continuous LNA mounting structure, a thermal short circuit formed between the cold head of refrigerator 1 and refrigerator 2, causing heat to flow along the copper LNA baseplate from the warmer side (refrigerator 2) to the cold head of refrigerator 1, resulting in a thermal back-flow phenomenon. Second, the LNAs and their mounting structures themselves constitute a large thermal load, requiring at least a corresponding refrigeration capacity (approximately 3.43 W) to maintain a low temperature. Since refrigerator 1 (refrigeration capacity 3.02 W) could not satisfy this demand on its own, it was also further dragged down by the weaker refrigerator 2 (refrigeration capacity 2.21 W) through thermal coupling, ultimately locking the temperature of the joint cryogenic structure at an equilibrium point between the two (22.3 K). The partition design not only severed the thermal coupling and thermal back-flow between the two refrigerators but also halved the thermal load connected to each refrigerator, thereby improving the overall refrigeration efficiency of the cryogenic system.

From the perspective of thermal analysis and refrigerator selection, once the components to be refrigerated and the structural design are determined, the purpose of thermal analysis is to determine the required refrigeration power based on the cryogenic structure. When the refrigeration capacity of a single refrigerator cannot meet the demand, multiple refrigerators can be employed to achieve a cumulative refrigeration power for the entire system. However, the prerequisite for such accumulation is that the components refrigerated by each refrigerator are thermally independent of one another. In this work, after implementing the partition design, the combined actual refrigeration capacity of the 20 K cold stages across the whole system (3.02 W + 2.21 W) exceeded 3.43 W, thereby enabling the LNA mounting structure to be refrigerated below 20 K. It should be noted that this cumulative effect refers not to the refrigeration power of any individual refrigerator after partitioning, but to the total refrigeration power available to the entire cryogenic system.

It should be noted that the LNAs were not powered during the cryogenic experiments in this study. Based on the single LNA power consumption provided by the manufacturer (30 mW), the total power consumption of the six LNAs is 0.18 W. Therefore, this additional load does not significantly affect the test results under the existing thermal load configuration (3.43 W). However, for a practical PAF receiver where all array outputs are equipped with LNAs, meaning at least 200 LNAs would be installed behind the array, the resulting power consumption would reach 6 W—a thermal load contribution that cannot be ignored [29]. Consequently, in addition to selecting LNAs with lower power consumption, it is essential in the cryogenic design process to choose an appropriate number of refrigerators and models with greater refrigeration capacity (e.g., the M1020 refrigerator) based on thermal analysis.

## 5. Conclusions

To further investigate the cryogenic technology for PAF receivers and to analyze and verify the refrigeration performance of different Dewar structures, this study conducted a full-array refrigeration design based on a PCB-based Vivaldi antenna array (developed by XAO) combined with six cryogenic LNAs. The Dewar structure includes a large-aperture microwave window, LNA assembly, two-stage cold transfer, as well as support and thermal insulation structures. Based on thermal analysis results, two M350 refrigerators were selected. An experiment with the two refrigerators operating jointly on the relevant components measured the temperature of the LNA mounting structure to be 22.3 K, which did not reach the target temperature below 20 K.

To analyze the current joint refrigeration scheme, we first conducted cryogenic experiments using each refrigerator individually on the LNA mounting structure, while keeping all other components inside the Dewar unchanged. The measured temperatures were 22.1 K and 22.7 K, respectively. Subsequently, by installing resistive heaters at each cold stage and recording their supply power, we measured the actual refrigeration capacities of the two refrigerators: 3.02 W @ 20 K and 18.4 W @ 77 K for refrigerator 1, and 2.21 W @ 20 K and 14.4 W @ 77 K for refrigerator 2. Through multi-point fitting, the refrigeration power demand of the LNA mounting structure was estimated to be approximately 3.43 W. Meanwhile, using another cryogenic test platform in the laboratory capable of providing 4 W @ 20 K, we conducted an additional cryogenic experiment on the same LNA mounting structure. The measured temperature was 14.5 K, confirming that the actual refrigeration demand of this structure is within 4 W.

Based on the analysis of the above experimental schemes and measured results, the key conclusion is that when two refrigerators are jointly connected to the LNA mounting baseplate to refrigerate the same structure, thermal coupling and thermal back-flow occur between them through the connecting structure, causing the refrigeration performance to settle at an intermediate state between the individual refrigeration results of the two refrigerators. To fully utilize the efficiency of each refrigerator, it is necessary to physically partition two refrigerators, thereby cutting off the crosstalk between two refrigerators and thereby improving the overall refrigeration efficiency of the cryogenic system. Guided by this conclusion, an optimization experiment was conducted in which the LNA mounting structures were divided into two equal parts and connected to the two refrigerators separately. The remeasured temperatures of the two parts reached 15.2 K and 18.2 K, respectively, both achieving the target below 20 K.

Meanwhile, the partition optimization scheme applied to the 20 K cold stage of multiple refrigerators described in this paper can also be extended to the 77 K cold stage of the array. In this case, to preserve the integrity of the PCB Vivaldi antenna array used in the experiment, we did not blindly adopt a cutting approach to conduct dedicated refrigeration experiments on the 77 K cold stage. Considering that the combined measured refrigeration capacity of the two refrigerators’ 77 K cold stage is sufficient to meet the refrigeration power after thermal analysis, the fact that the measured array temperature was only 165 K clearly indicates that the array was also affected by the joint structure. Regarding the metal array that we plan to adopt in the future, it is feasible to leave a certain partition gap without compromising microwave performance, thereby enabling the entire array to be refrigerated to the target temperature of 77 K using multiple refrigerators. This will be the focus of our next steps.

## Figures and Tables

**Table 1:** PAF receiver noise temperature calculation under the partial refrigeration scheme.

Noise Contribution	Physical Temperature	Comprehensive Transmission Parameters	Noise Temperature
Radome	295 K	−0.1 dB	7 K
Antenna Array	295 K	−0.4 dB	28.3 K
Coupling Noise	295 K	0.04	11.8 K
LNA	20 K	0.065 dB	4.5 K
Total			51.6 K

**Table 2:** PAF receiver noise temperature calculation under full-array refrigeration scheme.

Noise Contribution	Physical Temperature	Comprehensive Transmission Parameters	Noise Temperature
Vacuum Window	295 K	−0.1 dB	7 K
Antenna Array	77 K	−0.4 dB	7.4 K
Coupling Noise	77 K	0.04	3.1 K
LNA	20 K	0.065 dB	4.5 K
Total			22 K

**Table 3:** PAF receiver noise temperature calculation under the partition design.

Noise Contribution	Physical Temperature	Comprehensive Transmission Parameters	Noise Temperature
Vacuum Window	295 K	−0.1 dB	7 K
Antenna Array	165 K	−0.4 dB	15.9 K
Coupling Noise	165 K	0.04	6.6 K
LNA	16.5 K	0.061 dB	4.2 K
Total			33.7 K

## Data Availability

The data that support the findings of this study are available from the corresponding author upon reasonable request.
